# Psychometric properties of the Late-Life Function and Disability Instrument: a systematic review

**DOI:** 10.1186/1471-2318-14-12

**Published:** 2014-01-29

**Authors:** Marla K Beauchamp, Catherine T Schmidt, Mette M Pedersen, Jonathan F Bean, Alan M Jette

**Affiliations:** 1 Department of Physical Medicine and Rehabilitation, Harvard Medical School, Spaulding Outpatient Center Cambridge, Cambridge, Massachusetts, USA; 2 Massachusetts General Hospital Institute of Health Professions, Boston, Massachusetts, USA; 3 Clinical Research Center, Copenhagen University Hospital, Hvidovre, Denmark; 4 Health and Disability Research Institute, Boston University School of Public Health, Boston, Massachusetts, USA

**Keywords:** Function, Disability, Psychometric properties, Community-dwelling older adults

## Abstract

**Background:**

The choice of measure for use as a primary outcome in geriatric research is contingent upon the construct of interest and evidence for its psychometric properties. The Late-Life Function and Disability Instrument (LLFDI) has been widely used to assess functional limitations and disability in studies with older adults. The primary aim of this systematic review was to evaluate the current available evidence for the psychometric properties of the LLFDI.

**Methods:**

Published studies of any design reporting results based on administration of the original version of the LLFDI in community-dwelling older adults were identified after searches of 9 electronic databases. Data related to construct validity (convergent/divergent and known-groups validity), test-retest reliability and sensitivity to change were extracted. Effect sizes were calculated for within-group changes and summarized graphically.

**Results:**

Seventy-one studies including 17,301 older adults met inclusion criteria. Data supporting the convergent/divergent and known-groups validity for both the Function and Disability components were extracted from 30 and 18 studies, respectively. High test-retest reliability was found for the Function component, while results for the Disability component were more variable. Sensitivity to change of the LLFDI was confirmed based on findings from 25 studies. The basic lower extremity subscale and overall summary score of the Function component and limitation dimension of the Disability component were associated with the strongest relative effect sizes.

**Conclusions:**

There is extensive evidence to support the construct validity and sensitivity to change of the LLFDI among various clinical populations of community-dwelling older adults. Further work is needed on predictive validity and values for clinically important change. Findings from this review can be used to guide the selection of the most appropriate LLFDI subscale for use an outcome measure in geriatric research and practice.

## Background

Accurate assessment of physical functional limitations and disability is critical for improving access to health care services for older adults, and for evaluating the effectiveness of interventions designed to slow or prevent the progression of late-life disability [1,2]. Detecting meaningful changes in function and disability in older adults can be challenging, particularly if the outcome tool is not designed to accurately assess or reflect the purported change. The choice of outcome measure for use as a primary outcome in studies with older adults should be guided by the construct being measured and evidence for its psychometric properties [3].

Patient-reported measures (PROs) of function and disability are commonly used in studies of older adults because of their low cost and convenience. However, many existing measures were not designed for evaluative purposes and do not offer a comprehensive assessment of function or disability based on an explicit theoretical framework [4]. The Late-Life Function and Disability Instrument (LLFDI) was developed to overcome some of these limitations [5,6]. Unlike many other PROs, the LLFDI comprehensively assesses discrete functional tasks and operationalizes disability in important life roles beyond the narrow construct of activities of daily living.

The conceptual underpinnings for the LLFDI was Nagi’s disablement model [7] and also draws from the World Health Organization’s International Classification of Functioning, Disability, and Health (ICF) [8]. The LLFDI assesses both functional limitations (inability to perform discrete physical tasks) and disability (inability to participate in major life tasks and social roles). The Function component evaluates difficulty in performing 32 physical tasks and is comprised of an overall scale of function and three subscales: basic lower extremity, advanced lower extremity and upper extremity. The Disability component evaluates limitations in and frequency of taking part in 16 major life activities. The frequency dimension is comprised of social and personal role subscales plus an overall scale; the limitation dimension includes instrumental and management role subscales plus an overall scale. Raw scores are transformed to scaled scores (0–100) based on a Rasch model with higher scores indicating better levels of functioning.

Since its development in 2002, the LLFDI has been frequently used as an outcome measure in geriatric research. While the original LLFDI development papers [5,6] provide preliminary support for its validity and reliability, there is no synthesis of research on its psychometric properties. The objectives of this systematic review are to characterize the use of the LLFDI in published studies of community dwelling older adults and to evaluate the current available evidence on its psychometric properties.

## Methods

We conducted a systematic review of studies reporting results of the administration of the LLFDI in community-dwelling older adults. The methodology is based on PRISMA guidelines [9] for systematic reviews.

### Search strategy

Searches were performed by one investigator (MB) in consultation with a librarian. Study identification began with electronic searching of the ISI Web of Science for studies citing the two original LLFDI development papers [5,6]. We also searched the following electronic databases from inception until January 28th 2013: PubMed, Web of Science, CINAHL, PsychInfo, Google Scholar, JSTOR, ScienceDirect, WileyInterscience, and EMBASE. Key search terms were “Late Life Function and Disability Instrument”, “LLFDI” and “Late life FDI”. Finally, reference lists from relevant studies were hand-searched to ensure all possible studies were identified.

### Inclusion criteria

Two investigators (MB and CS) independently screened abstracts of retrieved papers with disagreements resolved by discussion. Full texts of relevant studies were then independently assessed by two reviewers (MB and CS) with disagreements resolved by consultation with a third reviewer (AJ). Inclusion criteria comprised:

•Types of studies: Any study design reporting results based on administration of the original version of the LLFDI.

•Types of participants: Studies including community-dwelling (non-institutionalized) older adults (mean age > 60 years).

Studies not published in English and conference abstracts were excluded.

### Data extraction

Two investigators (CS and MP) independently extracted data into a standardized form. The data extraction form was pilot tested prior to its use to ensure clarity and consistency. A third investigator (MB) reviewed and verified the extracted data for each study.

Data on background characteristics (participants, study purpose, sample size, design, scales reported) were extracted for each study. Thereafter, where available, data related to construct validity (convergent/divergent and known-groups), reliability (test-retest), and sensitivity to change (between-group results and within-group analyses) were extracted.

### Data synthesis

Data related to each psychometric property were summarized in tables. By convention, we interpreted a correlation coefficient of <0.3 as weak, 0.3 to 0.7 as moderate and >0.7 as strong. To facilitate synthesis of the sensitivity to change findings, where possible, we calculated Cohen’s effect sizes [10] (mean change/SD_baseline_) for within-group analyses. Graphs were created to visually depict the effect size results by scale. Values of 0.20, 0.50, and 0.80 have been used to represent small, moderate and large effect sizes, respectively [10].

## Results

### Search results

**Figure 1:**
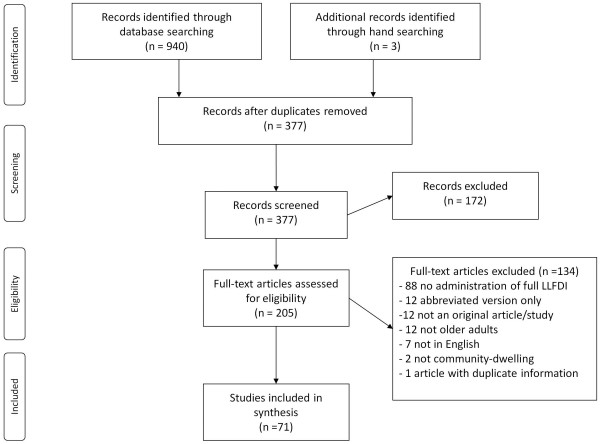
Study identification process.

The study selection process is outlined in Figure 1. Of a possible 940 studies, 71 were included [5,6,11-79]. Background characteristics of each study are summarized in Table S1 of Additional file 1. In total, the LLFDI was administered to 17,301 older adults with individual study sample sizes ranging from 11 [28] to 1,441 [27]. The majority of studies were conducted in the United States, however the LLFDI has also been used in Canada [21,22,24,32,48,58-60,64], Israel [37,51,52,72], Australia [17,23,29], New Zealand [39,67], Iceland [12,13], and the United Kingdom [24]. The study designs included cross-sectional, cohort and clinical trials. Many studies focused on community-dwelling older adults in general, however a wide range of specific older clinical populations were also represented including: pre-frail and mobility limited older adults [14,15,20,23,29,33,36,47],[66,67,70,75,76], various musculoskeletal populations (osteoarthritis, total joint replacement, fibromyalgia) [11,21,22,27,32,42,59,60],[65,69-71,74,78], cancer [24,48,55,58,79], psychological disorders (depression, anxiety) [38,46,61,73], stroke [18,45,57], veterans [18,54], urinary incontinence [37] and coronary heart disease [44]. The mean age across studies was 73 years (range 62 to 102). Most commonly, the overall function score of the Function component and limitation and frequency dimensions of the Disability component were used.

### Convergent/divergent validity

Data related to convergent/divergent validity of the LLFDI, that is, the degree to which LLFDI components and subscales correlated with measures of conceptually related (convergent) or unrelated (divergent) constructs, were extracted from 30 studies [12,13,15,17,25,27-29,32,33],[36-38,42,44,45,47,49,51,52],[56,61-63,65,66,68,71,72,74]. We hypothesized that moderate to strong correlations would be seen for variables theoretically related to function and disability (i.e., health status, function, mobility, balance and physical activity measures) while weak to moderate correlations would be observed for those variables less related theoretically to function and disability (e.g., biochemical markers). The correlation coefficients reported in the text below represent the range of coefficients observed between the various scales of the LLFDI and the related measure of interest. Detailed results for each individual study (correlation coefficients and statistical significance for each subscale) are outlined in Table S2 of Additional file 1.

#### Function component

The Function component of the LLFDI consistently demonstrated moderate to strong correlations with other self-report health-status and multi-component function scales including the 10-item Physical Functioning Scale of the SF-36 (PF-10) (r = 0.51 to 0.85) [25], Activities of Daily Living scale (r = −0.53 to −0.68) [28], Bradburn Affect Balance Scale (BABS) (r = 0.51 to 0.80) [28], Multidimensional Fatigue Inventory (MFI) (r = 0.46 to 0.64) [28], self-rated health (r = 0.68 to 0.70) [28], RAND-36 physical functioning subscale (r = 0.83) [44] and the London Handicap Scale (LHS) (r = 0.65) [44]. Moderate to strong correlations were also seen between LLFDI Function and single-concept mobility scales such as the Modified Gait Efficacy Scale (mGES) (r = 0.88) [56] and Physical Activity Scale for the Elderly (PASE) (r = 0.56) [44].

The LLFDI Function component demonstrated moderate to strong correlations with performance-based measures of multi-component function including the Short Physical Performance Battery (SPPB) (r = 0.29 to 0.67) [15,68,71] and Timed Up and Go (TUG) (r = −0.34 to −0.71) [51,52,66]. Moderate to strong correlations were also observed between LLFDI Function and single-concept performance-based mobility measures such as objectively measured physical activity (r = −0.30 to −0.70) [28], the Figure-of-8 Walk Test (F8W) (r = −0.45) [33], Berg Balance Scale (BBS) (r = 0.30 to 0.75) [51,52,66], walking speed (r = −0.55 to −0.57) [44], six-minute walking test (6MWT) (r = 0.62) [44], sit-to-stand test (r = −0.56) [44] and 400-meter walk (r = 0.26 to 0.73) [68,71].

In general, evidence for convergent validity was strongest for the overall function scale followed by the two lower-extremity sub-scales. The upper extremity sub-scale showed the lowest associations with other measures of function; however the latter primarily consisted of lower-extremity tasks. Evidence for divergent validity was shown by the weaker to moderate correlations found between the LLFDI Function component and less theoretically related constructs (neighbourhood walkability scores, Acylcarnitine factor scores, Vitamin D metabolites, B12, folate, Tangible Social Support Scale, age, BMI, income, education) [17,49,63,72,74].

#### Disability component

The Disability component demonstrated moderate correlations with other self-report health status and multi-component functional scales including the LHS (r = 0.47 to 0.66) [25,44], PF-10 (0.35 to 0.47) [25,38], Rand-36 physical functioning subscale (r = 0.38 to 0.68) [44], Hamilton Rating Scale for Depresssion-17 (r = −0.38) [38] and Anxiety [38,61] (r = −0.30 to −0.41), Western Ontario and McMasters Universities Osteoarthritis Index (WOMAC) (r = −0.23 to −0.47) [65] and the Center for Epidemiologic Studies Depression Scale (r = −0.38 to −0.56) [65]. Moderate to strong correlations were also seen between LLFDI Disability and single-concept mobility scales such as the PASE (r = 0.54 to 0.56) [44] and mGES (r = 0.32 to 0.63) [56].

Weak to moderate correlations were found between the Disability component and performance-based measures of multi-component function including the SPPB (r = 0.16 to 0.37) [68] and TUG (r = −0.06 to −0.30) [51,52]. Moderate to strong correlations were also observed between LLFDI Disability and single-concept performance-based mobility measures such as the F8W (r = −0.26) [33], BBS (r = 0.15 to 0.35) [51,52], walking speed (r = 0.01 to −0.33) [44], 20-meter walk (r = 0.24 to 0.37) [65] and 400-meter walk tests (r = 0.20 to 0.44) [68].

In general, the limitation dimension showed greater associations with the self-report and performance-based measures than the frequency dimension. Evidence for divergent validity was shown by the generally weak correlations between the LLFDI Disability component and less theoretically related constructs (neighbourhood walkability scores, Vitamin D metabolites, B12, folate, coping strategies, pain, body fat percentage, BMI) [17,27,37,65,72].

### Known-groups validity

Data related to know-groups validity of the LLFDI, that is, the degree to which scores of the Disability and Function components distinguished between groups known to differ, were extracted from 18 studies [5,6,27,29,30,36-38,40,47],[48,51,52,61,68,69,72,73] and are shown in Table 1. Discrimination between groups was considered if comparisons of the LLFDI between different subgroups of an independent measure or external parameter achieved statistical significance.

**Table 1:** Known-groups validity of the Late-Life Function and Disability Instrument

**Study**	**Scale(s)**	**Function component**	**Disability component**
Foster et al. 2011 [27]	Disability (IR)		Lower body obesity vs. central obesity group:
			No between-group differences in men or women for IR.
Gibson et al. 2010 [29]	Function (overall) Disability (FREQ, LIM)	Community dwellers vs. retirement dwellers and males vs. females: Overall function discriminated between both groups (p = 0.015 and p < 0.001, respectively).	Community dwellers vs. retirement dwellers:
			No between-group differences in FREQ or LIM.
			Males vs. females:
			FREQ (p = 0.013) discriminated between groups.
Gitlin et al. 2012 [30]	Function (overall)	Female vs. male and depressed vs. non-depressed:	
		Overall function differed in both groups (p < 0.01 and p < 0.001, respectively).	
Haley et al. 2002 [5]	Function (overall, UE, BLE, ALE)	Functional limitation groups measured by the PF-10:	
		Overall function and ALE discriminated between severe vs. moderate, moderate vs. slight and slight vs. none (all p < 0.0167). BLE and UE discriminated between severe vs. moderate and moderate vs. slight (all p < 0.0167).	
Jette et al. 2002 [6]	Disability (FREQ, LIM, SR, PR, IR, MR)		Functional limitation groups measured by the PF-10:
			FREQ, SR, LIM and IR all discriminated between severe vs. moderate, moderate vs. slight and slight vs. none groups (all p < 0.0167). PR discriminated between moderate vs. slight (p < 0.0167).
Julius et al. 2012 [36]	Function (overall, BLE, ALE) Disability (LIM)	No exertion during walking vs. some exertion during walking:	No exertion during walking vs. some exertion during walking:
		Overall function (p = 0.011), BLE (p = 0.012) and ALE (p = 0.022) all discriminated between groups.	LIM (p = 0.024) discriminated between groups.
Kafri et al. 2012 [37]	Function (overall, UE, BLE, ALE) Disability (LIM, FREQ, IR, MR, SR, PR)	Urgency urinary incontinence (UUI) vs. age-matched controls: Lower overall function (p < 0.001) and ALE (p < 0.001) in those with UUI.	Urgency urinary incontinence (UUI) vs. age-matched controls:
			No differences between groups in Disability.
Karp et al. 2009 [38]	Disability (LIM, FREQ)		Not depressed vs. depressed:
			Lower FREQ and LIM scores in depressed (both p < 0.001).
Kerr et al. 2012 [40]	Function (overall)	<30 min physical activity vs. 30 + min physical activity: Function differed between groups (p = 0.002). <30 min outdoors vs. 30+ min outdoors: Function differed between groups (p = 0.007).	
Li et al. 2012 [47]	Function (overall) Disability (LIM, FREQ)	High habitual gait speed (HGS) vs. moderate vs. low: Function (overall) (p < 0.001) discriminated between groups.	High habitual gait speed (HGS) vs. moderate vs. low: LIM (p < 0.001) discriminated between groups.
Lowe et al. 2009 [48]	Function (BLE, UE, ALE)	Walking ≥ 30 min/day vs. walking < 30 min/day and walking ≥ 60 min/day vs. walking < 60 min/day:	
		Trend for higher ALE in subjects who walked ≥ 30 min/day (p = 0.17) and in ≥ 60 min/day group (p = 0.10).	
Melzer & Kurz 2009 [51]	Function (BLE, UE, ALE) Disability (LIM, FREQ)	Non-fallers vs. one-time fallers vs. recurrent fallers:	Non-fallers vs. one-time fallers vs. recurrent fallers:
		Overall function (p = 0.04) and BLE (p = 0.03) discriminated between recurrent fallers and non-fallers.	No differences between groups.
Melzer et al. 2007 [52]	Function (overall, UE, BLE, ALE), Disability (FREQ, LIM, SR, PR, IR, MR)	Cane vs. non-cane (NC) users:	Cane vs. non-cane (NC) users:
			Overall function (p = 0.001), UE (p = 0.004), BLE (p = 0.001), ALE (p = 0.003) all discriminated between groups.	LIM (p < 0.001), IR (p < 0.001), MR (p = 0.001), FREQ (p = 0.047) and SR (p = 0.046) all discriminated between groups. Trend for a difference in PR (p = 0.16).
Porensky et al. 2009 [61]	Disability (FREQ, LIM)		Controls vs. those with Generalized Anxiety Disorder (GAD) without comorbitidy (CM) vs. those with GAD and CM:	
			Both FREQ and LIM discriminated across groups (p < 0.001). Post-hoc analysis showed FREQ and LIM differed in both GAD groups vs. controls.	
Sayers et al. 2004 [68]	Function (overall, UE, BLE, ALE) Disability (LIM, FREQ)	Mobility limited vs. non-mobility limited based on 400 m walk: Overall function, UE, BLE, ALE all discriminated between groups (all p < 0.01).	Mobility limited vs. non-mobility limited based on 400 m walk:	
			LIM and FREQ both discriminated between groups (both p < 0.01).	
Segal et al. 2013 [69]	Function (ALE)	High vs. moderate vs. low functioning based on chair-stand test:		
		Men in the high-functioning group reported higher ALE than those in the moderate and low-functioning groups (p = 0.004).		
Shahar et al. 2009 [72]	Function (overall, BLE) Disability (LIM)	Fallers vs. non-fallers:	Fallers vs. non-fallers:	
		Trend for difference in overall function (p = 0.06) and BLE (p = 0.16).	No difference in LIM between groups.	
Sriwattanakomen et al. 2010 [73]	Disability (LIM, FREQ)		Black vs. white:	
			LIM greater in blacks vs. whites (p = 0.02). FREQ not different between groups.	

*BLE*, Basic lower extremity scale; *ALE*, Advanced lower extremity scale; *UE*, Upper extremity scale; *FREQ*, Frequency dimension; *LIM*, Limitation dimension; *SR*, Social role domain; *PR*, Personal role domain; *IR*, Instrumental role domain; *MR*, Management role domain.

#### Function component

The LLFDI Function component discriminated between groups based on residence status [29], gender [30], depression [30], urinary incontinence [37], level of function and mobility limitation [5,68], physical activity levels [40], gait speed [47], fall status [51], walking exertion [36], cane use [52] and sit-to-stand performance [69]. Evidence for known-groups validity was strongest for the overall function score followed by the two lower-extremity scales.

#### Disability component

The Disability component of the LLFDI discriminated between groups based on gender [29], race [73], level of function and mobility limitation [5,68], depression [38], anxiety [61], cane use [52], gait speed [47] and walking exertion [36]. Unlike the Function component, the Disability component did not discriminate between groups based on residence status [29], urinary incontinence [37] or fall status [51]. Evidence for known-groups validity was strongest for the limitation dimension and associated instrumental role domain compared to the frequency dimension and associated domains.

### Reliability

Only three studies [5,6,52] included information related to the test-retest reliability of the LLFDI. Short-term stability of the English version of the LLFDI was only examined in the original development papers.

#### Function component

Intra-class correlation coefficients (ICCs) for the Function component were 0.96 for overall function, 0.97 for advanced lower-extremity, 0.98 for basic lower extremity and 0.91 for upper extremity (n = 15, 12-day testing interval) [5]. For the Hebrew version examined by Melzer et al. [52], test-retest ICCs were 0.9, 0.86, 0.77 and 0.79 for overall function, advanced/basic lower extremity and upper extremity scales, respectively (n = 55, 10–14 day test interval).

#### Disability component

Test-retest ICCs for the Disability component were 0.68 for the frequency dimension, 0.75 for the social role domain, 0.63 for the personal role domain, 0.82 for the limitation dimension, 0.83 for the instrumental role domain, and 0.44 for the management role domain (n = 15, 12 day interval) [6]. For the Hebrew version, ICCs were, 0.8, 0.83, 0.63, 0.69, 0.72 and 0.69 for each of the respective scales as listed above (n = 55, 10–14 day interval) [52].

### Sensitivity to change

Data on sensitivity to change were extracted from 25 studies [11,14,18-20,22-24,38,39,44,46],[53-55,57-59,64,66,67,70,75,76],[79]; 18 were RCTs [11,14,18-20,23,39,46,53-55,57],[64,66,67,75,76,79], 2 were cohort studies [22,59], 3 were single-group studies [38,58,70] and 1 was a cross-over trial [24]. One study was cross-sectional [44] but was included as it contained information on minimal detectable change (MDC). A detailed description of the individual study results is provided in Table S3 of Additional file 1. To facilitate interpretation of results, we classified studies as either 1) positive trials (i.e., RCTs in which there was a between-group difference in favor of the intervention in 1 primary or >2 secondary outcomes) 2) neutral trials or 3) single-group analyses (for cohort studies or single-group interventions). Among the 12 positive RCTs [14,18,19,39,46,53-55,57,75],[76,79], between-group differences in favor of the intervention group were detected by the LLFDI in 9 studies [18,19,39,46,53-55,57,76].

#### Function component

**Figure 2:**
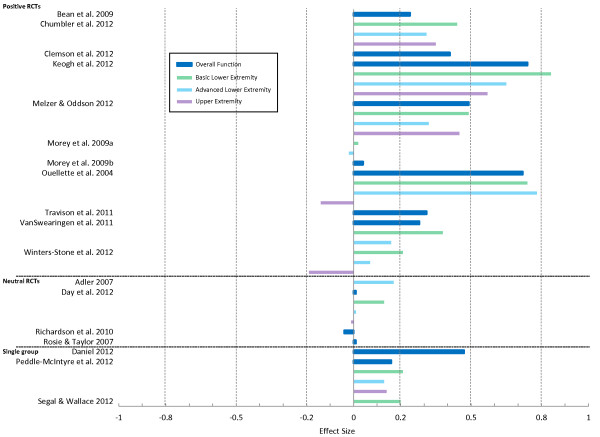
Effect sizes for the Function component of the Late Life Function and Disability Instrument.

A summary of the calculated effect sizes (ES) for the LLFDI Function component can be found in Figure 2. Eleven RCTs were classified as positive trials [14,18,19,39,53-55,57,75,76],[79]; all interventions included some type of exercise intervention with the exception of 1 trial [75] of testosterone administration. The basic lower extremity scale showed the greatest sensitivity to change (mean ES 0.45, range 0.02 to 0.84, n = 7) [18,39,53,55,57,76,79], followed by overall function (mean ES 0.40, range 0.04 to 0.74, n = 8) [14,19,39,53,54,57,75,76], advanced lower extremity (mean ES 0.33 range −0.02 to 0.78, n = 7) [18,39,53,55,57,76,79], and upper extremity (mean ES 0.21, range −0.19 to 0.57, n = 5) [18,39,53,57,79] scales (see Table 2). Of note, in the positive Morey et al. trial [55], although the ES for basic and advanced lower extremity scales in the intervention group were negligible (0.02 and −0.02, respectively), results were favourable against the backdrop of functional decline in the control group. Among the neutral trials [11,23,64,67] (n = 4), ES estimates ranged from −0.04 [64] to 0.17 [11]. Within the single-group analyses [20,58,70] (n = 3), a Wii-Fit rehabilitation program [20] was associated with the greatest ES for overall function (0.47), while ES after a resistance training program among cancer survivors [58] ranged from 0.13 (advanced lower extremity) to 0.21 (basic lower extremity), and was 0.20 for basic lower extremity after an aquatic power training program [70].

**Table 2:** Summary of mean effect sizes for the Late-Life Function and Disability Instrument in positive randomized controlled trials

**Component**	**Subscale**	**Mean effect size (range)**	**No of studies, total sample size**
Function	Overall	0.40 (0.04 to 0.74)	8, 487
	Basic lower extremity	0.45 (0.02 to 0.84)	7, 466
	Advanced lower extremity	0.33 (−0.02 to 0.78)	7, 466
	Upper extremity	0.21 (−0.19 to 0.57)	5, 124
Disability	Limitation dimension	0.35 (−0.10 to 1.2)	7, 444
	Instrumental role	0.83 (0.47 to 1.28)	3, 66
	Management role	0.55 (0.48 to 0.62)	2, 43
	Frequency dimension	0.32 (0.13 to 0.67)	4, 300
	Social role	0.36 (0.01 to 0.71)	2, 43
	Personal role	0.30 (0.19 to 0.40)	2, 43

Information on meaningful change was available from two studies. In a cross-sectional study of older adults with chronic heart failure [44] the MDC_95_ was estimated at 4.3 points for overall function. In the 6-month RCT of testosterone administration in older men with mobility limitation [75], the minimal important difference for overall function (calculated using patient-reported global rating of change) was 2.7 points.

#### Disability component

**Figure 3:**
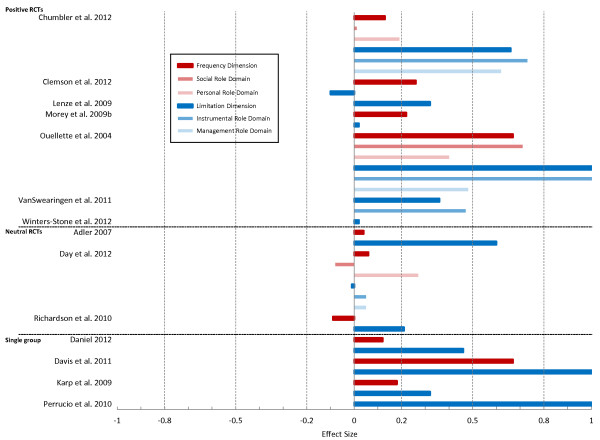
Effect sizes for the Disability component of the Late Life Function and Disability Instrument.

Figure 3 shows a summary of effect sizes for the Disability component. Seven RCTs were classified as positive trials [18,19,46,54,57,76,79]; 6 included some form of exercise intervention and 1 [46] was a trial of antidepressant therapy. The limitation dimension was associated a higher ES (mean ES 0.35, range −0.10 to 1.2, n = 7) [18,19,46,54,57,76,79] than the frequency dimension (mean ES 0.32, range 0.13 to 0.67, n = 4) (see Table 2) [18,19,54,57]. Among the domain roles, the highest ES was for instrumental (mean ES 0.83, range 0.47 to 1.28, n = 3) [18,57,76], followed by management (mean ES 0.55, range 0.48 to 0.62, n = 2) [18,57], social (mean ES 0.36 range 0.01 to 0.71, n = 2) [18,57] and personal (mean ES 0.30, range 0.19 to 0.40, n = 2) [18,57] roles. Effect sizes for the neutral RCTs [11,23,64] (n = 3) were all <0.30 except for the limitation dimension in the Tai Chi trial [11] (ES 0.60). For the single-group analyses [20,22,38,59] (n = 4), ES ranged from 0.12 (frequency dimension) and 0.46 (limitation dimension) after a Wii-fit rehabilitation intervention [20] to 0.67 (frequency dimension) and 1.6 (limitation dimension) in the cohort study of joint replacement surgery [22].

Information on meaningful change was only available from 1 study. In the cross-sectional study of older adults with chronic heart failure [44] the MDC_95_ was estimated at 7.8 points for the frequency dimension and 16.7 for the limitation dimension.

## Discussion

Since its conception in 2002, the LLFDI has been used as an outcome measure in over 70 studies including more than 17,000 community-dwelling older adults. Evidence for its psychometric properties has been demonstrated across a wide range of older clinical populations and contexts. The choice of LLFDI sub-scale for use in individual studies should depend on the construct of interest and evidence for relevant psychometric properties in the most applicable population. Results of this review can be used by researchers to guide future decisions regarding the use of the LLFDI as an outcome measure for clinical research in community-dwelling older adults.

The construct validity of both the Function and Disability components of the LLFDI was well-supported by the evidence found in this review. We noted moderate to strong convergent validity between the Function component and well-validated self-report and performance-based measures of function such as the PF-10 and SPPB. In addition, while there is no accepted gold-standard measure of disability, the Disability component was moderately associated with general health status measures such as the LHS and RAND-36 as well as with many commonly used self-report and performance-based measures of function. The LLFDI also showed strong known-groups validity with both components discriminating between groups based on various functional, demographic and medical categories. Our review did not reveal any studies evaluating the use of LLFDI measures of Function or Disability for predicting institutionalization or mortality, highlighting the need for further research on the predictive validity of the LLFDI.

Only three studies [5,6,52] investigated the test-retest reliability of the LLFDI and two were the original development papers. While very high reliability scores (ICCs 0.91-0.98) were reported for all Function scales, a wider range of reliability was reported within the Disability component (ICCs 0.44-0.82). In general, the Disability limitation and frequency dimensions showed moderate to high test-retest reliability with the limitation dimension and instrumental role domain showing the best reproducibility. The management role domain had the lowest reliability, likely due to the limited 4-item pool of this scale. Larger studies on test-retest reliability of the LLFDI would be helpful, especially in light of the lower reproducibility reported for the Disability component.

PROs are often thought to have limited capacity for detecting change given their breadth of measurement and vulnerability to external influences [1,80,81]. In this review, sensitivity to change of the LLFDI was confirmed based on findings from 25 studies. Most scales demonstrated small to moderate effect sizes in positive trials and in cohort studies in which the participants underwent a change in health status. In particular, we noted larger effect sizes for the basic lower extremity scale and summary score of the Function component as well as for the limitation dimension of the Disability component as compared to the other LLFDI scales. These results should be considered when selecting the most appropriate scale for use in clinical trials and longitudinal studies with community dwelling older adults. Only one study [75] attempted to define a clinically meaningful difference for the LLFDI, however this study included only men was based on a testosterone intervention. There remains a need for further work to determine the increments of change on the LLFDI that are clinically meaningful.

Our findings are subject to several limitations. A quality assessment was beyond the scope of this review and very few studies were designed specifically to measure psychometric properties of the LLFDI. We were unable to perform any formal meta-analysis due to the heterogeneity in study outcomes, clinical populations and design. While every attempt was made to identify relevant studies, it is possible that some studies were missed. Finally, our results are only applicable to the original version of the LLFDI administered in community-dwelling older adults. An abbreviated version of the instrument [50] has been developed as well as a computer adaptive version [82] and the psychometric properties of these instruments should be considered separately.

## Conclusions

In summary, we have conducted a systematic review of the use of the LLFDI and evidence for its psychometric properties based on 71 published studies. While we have shown extensive data supporting the instrument’s construct validity and sensitivity to change among various clinical populations of community-dwelling older adults, further work is needed to determine the LLFDI’s predictive validity and values for clinically meaningful change. Results from this review can be used to inform the selection of the most appropriate LLFDI component and subscale for use as an outcome measure in geriatric research.

## Abbreviations

LLFDI: Late-life function and disability instrument; PRO: Patient-reported outcome; ES: Effect size; MDC: Minimal detectable change; ICC: Intra-class correlation coefficient; PF-10: Physical functioning scale of the SF-36; MGES: Modified gait efficacy scale; PASE: Physical activity scale for the elderly; BABS: Bradburn affect balance scale; MFI: Multidimensional fatigue inventory; LHS: London handicap scale; F8W: Figure-of-8 walk test; BBS: Berg balance scale; TUG: Timed up and go; 6MWT: Six-minute walking test; SPPB: Short physical performance battery; WOMAC: Western Ontario and McMasters Universities Osteoarthritis Index.

## Competing interests

AMJ has stock holdings in CREcare, LLC, a small business created to disseminate outcome instruments such as the LLFDI.

## Authors’ contributions

MKB was responsible for the conception, design and coordination of the study, data acquisition and interpretation, and drafting and revising the manuscript. CTS contributed to the acquisition of data and revision of the manuscript. MMP contributed to the acquisition of data and revision of the manuscript. JFB contributed to the general supervision of the study, conception, design, interpretation of data and revision of the manuscript. AMJ contributed to the general supervision of the study, conception, design, interpretation of data and revision of the manuscript. All authors approved the final manuscript.

## Pre-publication history

The pre-publication history for this paper can be accessed here:

http://www.biomedcentral.com/1471-2318/14/12/prepub

## Supplementary Material

Additional file 1 **Outlines data extraction results for each study in Tables S1, S2 and S3 as per below. Table S1.** Characteristics of studies reporting results based on the administration of the Late Life Function and Disability Instrument. **Table S2.** Convergent/divergent validity of the Late-Life Function and Disability Instrument. **Table S3.** Sensitivity to change of the Late-Life Function and Disability Instrument. Click here for file
