# Supplementary material for: Psychometric properties of the Late-Life Function and Disability Instrument: a systematic review
Source: BMC Geriatr. 2014 Jan 29;14:12. doi: 10.1186/1471-2318-14-12 (PMC3909447; doi:10.1186/1471-2318-14-12)
Supplement: Additional file 1 — Outlines data extraction results for each study in Tables S1, S2 and S3 as per below. Table S1. Characteristics of studies reporting results based on the administration of the Late Life Function and Disability Instrument. Table S2. Convergent/divergent validity of the Late-Life Function and Disability Instrument. Table S3. Sensitivity to change of the Late-Life Function and Disability Instrument. [file 1471-2318-14-12-S1.doc]

**ADDITIONAL FILE 1**

**Table S1. Characteristics of studies reporting results based on the administration of the Late-Life Function and Disability Instrument**

| **Study** | **Country** | **Population** | **Study purpose** | **n** | **Men**  **(n,%)** | **Age (mean±SD) yrs** | **Design** | **Scales reported** |
| --- | --- | --- | --- | --- | --- | --- | --- | --- |
| **Adler 2007** | USA | Elders with lower extremity joint pain from osteoarthritis | To assess the effects of Tai Chi on improving impairments, functional limitations and disability | 14 | 1,7 | 72±7 | RCT | Function  (ALE)  Disability  (LIM, FREQ) |
| **Arnadottir et al. 2011a** | Iceland | Older adults | To determine the relationship between components of the ICF and participation frequency and restrictions | 186 | 97,52 | 74±6 | Cross-sectional | Disability  (LIM, FREQ)  Function  (UE, ALE) |
| **Arnadottir et al. 2011b** | Iceland | Older adults | To identify determinants of high self-rated health | 185 | 96,52 | 74±6 | Cross-sectional | Function  (BLE, UE, ALE)  Disability  (LIM, FREQ) |
| **Bean et al. 2009** | USA | Mobility-limited older adults | To compare the NIA’s Advocated Progressive Resistance Training Program vs. the Increased Velocity Exercise Specific to Task program on muscle power and functional performance | 138 | 43,31 | 75±7 | RCT | Function  (overall) |
| **Bean et al. 2011** | USA | Mobility-limited older adults | To compare the association of physiological, psychosocial and health factors between the Short Physical Performance Battery and LLFDI | 137 | 42,31 | 75±7 | Cross-sectional | Function  (overall) |
| **Buman et al.**  **2010** | USA | Older adults | To examine the association between physical activity of different intensities and health and well-being | 862 | 379,44 | 76±7 | Cohort | Function  (ALE) |
| **Byles et al.**  **2012** | Australia | Older adults | To identify the extent to which homes and neighbourhoods are supportive | 260 | 128,49 | 77 | Cross-sectional | Function  (BLE, UE, ALE)  Disability  (LIM, FREQ, SR, IR, MR) |
| **Chumbler et al. 2012** | USA | Veterans post-stroke | To determine the effect of a multifaceted stroke telerehabilitation intervention on function and disability | 48 | 47,98 | 67±10 | RCT | Function  (BLE, UE, ALE)  Disability  (LIM, FREQ, SR, PR, IR, MR) |
| **Clemson et al. 2012** | Australia | Older adults with high fall risk | To determine if a lifestyle approach to balance and strength training reduces rate of falls | 317 | 174,55 | 83±4 | RCT | Function  (overall)  Disability  (LIM, FREQ) |
| **Daniel 2012** | USA | Pre-frail older adults | To compare the effects of Wii-Hab vs. seated exercise for decreasing indices related to frailty | 23 | 9,39 | 77±5 | Pilot RCT | Function  (overall)  Disability  (LIM, FREQ) |
| **Davis et al. 2011** | Canada | Patients undergoing total hip or knee replacement | To describe the trajectory of symptoms, daily activities, and participation and to evaluate the inter-relationships within and across time during first year following surgery | 931 | 372,40 | 64±11 | Cohort | Disability  (FREQ, LIM) |
| **Davis et al. 2012** | Canada | Patients undergoing total hip or knee replacement | To test the temporal relationship of body structure and function, activity and participation in the context of a person’s social and physical environment | 931 | 372,40 | 64±11 | Cohort | Disability  (LIM, FREQ) |
| **Day et al. 2012** | Australia | Pre-clinically disabled older adults | To test the effect of Tai Chi on progression of preclinical disability compared to seated flexibility exercise | 503 | 183,36 | >70 | RCT | Function  (overall, BLE, UE, ALE)  Disability  (LIM, FREQ, SR, PR, IR, MR) |
| **Demark-Wahnefried et al. 2012** | USA, Canada, UK | Cancer survivors | To determine adherence and long-term health outcomes of a home-based diet and exercise intervention | 488 | 218,45 | 73±5 | Cross-over trial | Function  (BLE, ALE) |
| **Dubuc et al. 2004a** | USA | Older adults | To determine the effects of using questions with and without health attribution on scores derived from the LLFDI | 75 | 18, 24 | 77±9 | Cross-sectional | Function  (BLE, ALE, UE)  Disability  (LIM, FREQ) |
| **Dubuc et al. 2004b** | USA | Older adults | To evaluate the concurrent validity, comprehensiveness and precision of the LLFDI compared to other measures | 75 | 18,24 | 77±9 | Cross-sectional | Function  (BLE, ALE, UE)  Disability  (LIM, FREQ) |
| **Foster et al. 2010** | USA | Obese adults at high risk for or already with knee osteoarthritis | To determine whether an association exists between fat distribution and lower limb physical function and disability | 1441 | 549,38 | 62±8 | Cross-sectional | Disability  (IR) |
| **Franke et al. 2012** | USA | Centenarians | To assess the association between perceived health, fatigue, positive and negative affect, handgrip strength, physical activity, BMI, and self-reported functional limitations | 11 | - | 102±1 | Cohort | Function  (overall, UE, BLE, ALE) |
| **Gibson et al. 2010** | Australia | Pre-clinically disabled older adults | To describe the use of a pre-clinical disability screening tool to select a functionally comparable group of older adults from different settings with early functional limitation and explore factors associated with function and disability | 471 | 151,32 | >70 | Cross-sectional | Function  (overall)  Disability  (FREQ, LIM) |
| **Gitlin et al. 2012** | USA | African American senior center members | To examine differences between depressed and non-depressed African American men and women | 153 | 56,37 | 73±8 | Cross-sectional | Function  (overall) |
| **Haley et al. 2002a** | USA | Older adults | To compare items on the SF-36 10-item physical functioning scale and LLFDI function component | 50 | 14,28 | 77±7 | Cross-sectional | Function  (overall) |
| **Haley et al.**  **2002b** | USA | Older adults | To report on the development and results of the initial field test of the LLFDI Function component | 150 | 35,23 | 76±9 | Cross-sectional | Function  (overall, ALE, BLE, UE) |
| **Hawker et al. 2008** | Canada | Adults with hip and knee osteoarthritis (OA) | To evaluate the preliminary psychometric properties of a new OA pain measure | 100 | 22,22 | 75 (range 51-93) | Cross-sectional | Disability  (LIM) |
| **Hess et al. 2010** | USA | Older adults with mobility disability | To validate the Figure-of-8 Walk Test | 51 | 17,33 | 77±6 | Cross-sectional | Function  (overall)  Disability  (LIM) |
| **Hsu et al. 2010** | USA | Sedentary older adults | To examine the measurement properties of the LLFDI using data from the Lifestyle Interventions and Independence for Elders Pilot study | 424 | 132,31 | 70-89 | Cohort | Disability  (LIM) |
| **Jette et al. 2002** | USA | Older adults | To report on the development and results of the initial field test of the LLFDI Disability component | 150 | 35,23 | 76±9 | Cross-sectional | Disability  (LIM, FREQ, SR, PR, IR, MR) |
| **Jette et al. 2003** | USA | Older adults | To test the hypothesis that distinct Activity and Participation dimensions of the ICF could be identified using physical functioning items drawn from the LLFDI | 150 | 35,23 | 76±9 | Cross-sectional | Function  Disability |
| **Julius et al. 2012** | USA | Older adults with mobility limitations | To examine the relationship of perceived effort of walking with gait, function, activity, fear of falling, and confidence in walking | 50 | 17,34 | 77±6 | Cross-sectional | Function  (overall, BLE, ALE)  Disability  (LIM) |
| **Kafri et al. 2012** | Israel | Women with urgency urinary incontinence | To compare self-reported function and disability in women with urgency urinary incontinence vs. healthy controls | 132 | 0,0 | 62±6 | Cross-sectional | Function  (overall, UE, BLE, ALE)  Disability  (LIM, FREQ, IR, MR, SR, PR) |
| **Karp et al. 2009** | USA | Subjects experiencing a major depressive episode and depression-free older adults | To determine: the extent of disability in subjects with and without depression; the correlation between disability and depression severity and quality of life; disability response after antidepressant pharmacotherapy | 313 | 94,30 | 74±7 | Open-label trial | Disability  (LIM, FREQ) |
| **Keogh et al. 2012** | New Zealand | Older adults living in retirement villages | To determine the effects of 12 wks of dance (1x/wk & 2x/wk) on functional performance and physical activity | 45 | 3,6 | 78±6 | RCT | Function  (overall, UE, BLE, ALE) |
| **Kerr et al. 2012a** | USA | Older adults in retirement communities | To assess the relationship between time spent in physical activity, outdoor time and various health outcomes | 117 | 36,31 | 83±7 | Cohort | Function  (overall) |
| **Kerr et al. 2012b** | USA | Ambulatory older adults | To investigate physical activity locations and their relation to objectively measured physical activity and self rated health | 754 | 337,45 | 75±7 | Cross-sectional | Function  (ALE) |
| **Keysor et al. 2010** | USA | Older adults with or at risk of developing knee osteoarthritis | To identify the prevalence of mobility barriers and transportation facilitators and to examine association with disability | 438 | 134,30 | 70±4 | Cohort | Disability  (LIM, FREQ) |
| **King et al. 2011** | USA | Older adults | To evaluate the relations among objectively measured neighbourhood design, mobility impairment, physical activity and body weight | 719 | 338,47 | 74±6 | Cohort | Function  (ALE) |
| **LaPier 2012** | USA | Older adults with coronary heart disease | To examine the concurrent validity of the LLFDI and accuracy of information obtained through self-report questionnaire vs. interview | 29 | 21,72 | 69±9 | Cross-sectional | Function  (overall, UE, BLE, ALE)  Disability  (LIM, IR, MR, FREQ, SR, PR) |
| **LeBrasseur et al. 2006** | USA | Adults with a single ischemic stroke in past 6-24 months | To quantify the relationship among impairments in lower-extremity strength and power, measures of lower-extremity function and global disability | 31 | 23,74 | 66±8 | Cross-sectional | Disability  (LIM, IR, MR) |
| **Lenze et al. 2009** | USA | Older adults with generalized anxiety disorder | To examine the efficacy, safety and tolerability of the selective serotonin reuptake inhibitor (SSRI) escitalopram | 177 | 58,33 | 71±7 | RCT | Disability  (LIM) |
| **Li et al. 2012** | USA | Mobility-limited older adults | To evaluate the content validity of a customized exercise tolerance testing protocol | 137 | 44,32 | 75±7 | Cross-sectional | Function  (overall)  Disability  (LIM, FREQ) |
| **Lowe et al. 2009** | Canada | Palliative cancer patients | To examine the association between physical activity and quality of life | 50 | 20,40 | 62±13 | Cross-sectional | Function  (BLE, UE, ALE) |
| **Lum et al. 2011** | USA | Men not engaged in regular physical activity | To determine the association between physical function and plasma metabolites | 77 | 77,100 | 79±5 | Cross-sectional | Function  (overall, UE, BLE, ALE) |
| **McAuley et al. 2005** | USA | Older black and white women | To examine the psychometric properties of the LLFDI  ** Development of the abbreviated version of the LLFDI* | 249 | 0,0 | 68 (range 59-84) | Cross-sectional | Function  (ALE, BLE, UE), Disability  (FREQ, SR, PR, IR, MR) |
| **Melzer & Kurz 2009** | Israel | Healthy older adults from senior living residences | To compare self-reported function and disability between fallers and non-fallers | 100 | 27,27 | 78±6 | Cross-sectional | Function  (overall, UE, BLE, ALE)  Disability  (LIM, FREQ) |
| **Melzer & Oddsson 2012** | USA | Older adults | To evaluate a group-based balance program on balance function | 66 | 17,26 | 77±7 | RCT | Function  (BLE, UE, ALE) |
| **Melzer et al. 2007** | Israel | Non-disabled older adults | To investigate test-retest reliability, construct validity and known-groups validity of the Hebrew version of LLFDI | 55 | 13,24 | 80±5 | Cross-sectional | Function  (overall, UE, BLE, ALE)  Disability  (FREQ, LIM, SR, PR, IR, MR) |
| **Morey et al. 2009a** | USA | Overweight, long-term cancer survivors | To determine the effect of a home-based diet and exercise intervention on reorienting the functional decline | 641 | 302,47 | 73±5 | RCT | Function  (BLE, ALE) |
| **Morey et al. 2009b** | USA | Veterans | To determine the effects of physical activity counseling on measures of physical activity and function | 398 | 100,40 | 78±5 | RCT | Function  (overall)  Disability  (FREQ, LIM) |
| **Newell et al. 2012** | USA | Older adults | To determine the reliability, internal consistency and validity of the modified gait efficacy scale (mGES) | 102 | 26,26 | 79±6 | Cross-sectional | Function  (overall)  Disability  (FREQ, LIM) |
| **Ouellette et al. 2004** | USA | Older long-term stroke survivors | To determine the effects of high-intensity resistance training on strength, function and disability | 42 | 28,67 | 66±2 | RCT | Function  (overall, UE, BLE, ALE) Disability  (FREQ, SR, PR, LIM, IR, MR) |
| **Peddle-McIntyre et al. 2012** | Canada | Lung cancer survivors post- treatment | To evaluate the feasibility and preliminary efficacy of progressive resistance training | 17 | 7,41 | 67 (range 50-85) | Single-group feasibility study | Function  (ALE, BLE, UE) |
| **Perruccio et al. 2010** | Canada | Patients undergoing primary hip or knee replacement | To determine whether the distinct interpretations of self-rated-health could be identified and characterized in individuals undergoing health status changes | 449 | 179,40 | 64 (range 31-88) | Cohort | Disability  (LIM) |
| **Perruccio et al. 2011** | Canada | Patients undergoing total hip or knee replacement | To identify determinants of total joint replacement outcomes and identify areas for targeted intervention beyond physical aspects of health | 529 | 212,40 | 64 (range 31-88) | Cohort | Disability  (LIM) |
| **Porensky et al. 2009** | USA | Older adults with Generalized Anxiety Disorder (GAD) and healthy controls | To examine the burden of late-life GAD in terms of disability, health-related quality of life and health service utilization | 164 GAD  42 controls | 65,32 | 72±8 | Cross-sectional | Disability  (FREQ, LIM) |
| **Puthoff & Nielsen 2006** | USA | Older adults | To examine relationships between impairments in lower extremity muscle performance and functional limitations and disability | 30 | 5,17 | 77±7 | Cross-sectional | Function  (overall)  Disability  (LIM) |
| **Rancourt 2009** | USA | Older adults | To examine the relationship between flexibility and ability to perform activities of daily living | 32 | 3,10 | 72±6 | Cross-sectional | Function  (overall, UE, BLE, ALE)  Disability  (FREQ, SR, PR, LIM, IR, MR) |
| **Richardson et al. 2010** | Canada | People with a chronic illness | To determine the effect of rehabilitation offered within primary care on health status and hospital and emergency room visits | 303 | 111,37 | 45-95  (range) | RCT | Function  (overall)  Disability  (FREQ, LIM) |
| **Riddle & Jensen 2013** | USA | People with chronic knee osteoarthritis pain | To examine the construct and criterion-based validity of the 2 item per scale version of the Coping Strategies Questionnaire | 873 | 358,41 | 66±9 | Cohort | Disability  (FREQ, LIM) |
| **Ritchie**  **2009** | USA | Older adults with mild mobility impairment from a retirement community | To determine the effect of dose (1x/wk vs. 2x/wk) of the FallProof intervention | 23 | 1,4 | 79±7 | RCT | Function  (overall) |
| **Rosie & Taylor 2007** | New Zealand | Mobility limited older adults | To compare 6 weeks of daily home repeated sit-to-stand with progressive resistance knee extension using ankle weights | 66 | 19,29 | 85±4 | RCT | Function  (overall) |
| **Sayers et al.**  **2004** | USA | Ambulatory community-dwelling older adults | Evaluate concurrent and predictive validity of the LLFDI vs. performance-based measures of function | 101 | 37,37 | 81±4 | Cross-sectional | Function  (UE, BLE, ALE)  Disability  (FREQ, LIM) |
| **Segal et al.**  **2013** | USA | Adults with radiographic knee OA and daily symptoms | To determine which lower limb strength and joint kinetic and kinematic parameters distinguish sit-to-stand (STS) performance | 49 | 26,53 | 65±8 | Cross-sectional | Function  (ALE) |
| **Segal & Wallace**  **2012** | USA | Adults with symptomatic knee OA and mobility limitation | To determine the tolerance and feasibility of aquatic-based power training for improving lower limb muscle power, impairments and mobility | 29 | 11,38 | 67±9 | Single-group study | Function  (BLE) |
| **Segal et al.**  **2009** | USA | Adults with symptomatic knee OA | To assess whether patterns of movement can distinguish those with more severe mobility limitation from those without | 60 | 27,45 | 64±7 | Cross-sectional | Function  (ALE) |
| **Shahar et al.**  **2009** | Israel | Healthy older adults | To investigate the impact of essential nutritional elements on falls | 100 | 27,27 | 78±6 | Cross-sectional | Function  (overall, BLE)  Disability  (LIM) |
| **Sriwattanakomen et al.**  **2010** | USA | Subsyndromally depressed older African Americans | To compare frequencies of risk factors for depression between depressed older blacks and whites | 215 | 75,35 | 66±11 | Cross-sectional | Disability  (FREQ, LIM) |
| **Torma et al.**  **2013** | USA | Older adults with fibromyalgia | To identify predictors of physical function and to examine the influence of resilience on the relationship between pain and function | 224 | 13,6 | 62±7 | Cross-sectional | Function  (overall) |
| **Travison et al.**  **2011** | USA | Older men with mobility limitation and low total or free testosterone levels | To evaluate the clinical meaningfulness of the effects of testosterone therapy on performance-based and self-reported measures of muscle and function | 165 | 165,100 | 74±5 | RCT | Function  (overall) |
| **Van Swearingen et al.**  **2011** | USA | Older adults with slow and variable gait | To compare task-oriented, motor sequence learning exercise vs. impairment-oriented exercise on activity and participation outcomes | 47 | 17,35 | 77±6 | RCT | Function  (overall, BLE, ALE)  Disability  (LIM, IR) |
| **Vaughan & Giovanello 2010** | USA | Older adults | To examine relationships between 3 executive processes underlying executive function and 2 types of instrumental activities of daily living | 95 | 42,44 | 74±6 | Cross-sectional | Disability  (FREQ) |
| **White et al. 2010** | USA | Older adults with or at risk of knee osteoarthritis | To explore the association of features of a person’s neighbourhood environment with disability in daily activities | 436 | 135,31 | 70±4 | Cohort | Disability  (FREQ, LIM) |
| **Winters-Stone et al. 2012** | USA | Early stage, postmenopausal breast cancer survivors | To determine the effect of resistance and impact training on muscle strength and physical function | 106 | 0,0 | 62±7 | RCT | Function  (UE, BLE, ALE)  Disability  (LIM) |

BLE= basic lower extremity scale; ALE=advanced lower extremity scale; UE=upper extremity scale; FREQ=frequency dimension; LIM=limitation dimension; SR=social role domain; PR=personal role domain; IR= instrumental role domain; MR=management role domain

**Table S2.** **Convergent/divergent validity of the Late-Life Function and Disability Instrument**

| **Study** | **Scale(s)** | **Compared measure(s)** | **Correlation coefficients* or regression results** |
| --- | --- | --- | --- |
| **Arnadottir et al. 2011a** | Disability  (LIM, FREQ)  Function  (UE, ALE) | Components of ICF (Personal Factors, Environmental Factors, Body Functions, Activities) | FREQ (Beta) and:  Personal factors: Age (-0.31)*, Gender (0.16), Education (0.22)*, Recurrent fall history (-3.88)*, Physical Activities Scale for the Elderly (6.93)*, Medical diagnoses (-0.70)*.  Environmental Factors: Residency (3.51)*, Living alone (-0.27), Sufficient income (-1.06), Employed (0.43).  Body Functions: Activities-specific Balance Confidence Scale (0.67)*, Mini-Mental State Examination (0.80)*, Geriatric Depression Scale (-0.51)*, BMI (0.23), Bodily pain (0.02)*.  Activities: UE (0.05), ALE (0.10)*, Timed Up and Go (-0.48)*, Drives a car (3.65)*.  LIM (OR) and:  Personal factors: Age (0.95)*, Gender (1.98)*, Education (1.00), Recurrent falls (0.10)*, Physical Activities Scale for the Elderly (6.77)*, Medical diagnoses (0.58)*.  Environmental factors: Residency (4.83)*, Living alone (1.46), Sufficient income (0.79), Employed (2.22).  Body functions: Activities-specific Balance Confidence Scale (1.57)*, Mini-Mental State Examination (1.12), Geriatric Depression Scale (0.75)*, BMI (0.99), Bodily pain (1.02)*.  Activities: UE (1.07)*, ALE (1.08)*, Timed Up and Go (0.64)*, Drives a car (2.33)*. |
| **Arnadottir et al. 2011b** | Function  (BLE, UE, ALE)  Disability  (LIM, FREQ) | Self-rated health | Self-rated health and: BLE (OR=1.07)*; ALE (OR=1.06)*; UE (OR=1.07)*; FREQ (OR=1.13)*; LIM (OR=6.66)* |
| **Bean et al. 2011** | Function (overall) | Leg velocity, exercise tolerance test (ETT), chronic conditions, sex, falls efficacy, Short Physical Performance Battery (SPPB) | Model including leg velocity*, ETT duration*, chronic conditions*, female sex* and falls efficacy predicted overall Function with r^2^=0.42*  Overall function and: SPPB r=0.34* |
| **Byles et al. 2012** | Function  (BLE, UE, ALE)  Disability  (LIM, FREQ, SR, IR, MR) | Neighbourhood Environment Walkability Scale (NEWS), Home Falls Accident Screening Tool (HOME FAST), Neighbourhood Satisfaction and Safety Scales | FREQ and: Usability A&B r=0.2*, NEWS C r=0.2*, Neighbourhood Satisfaction r=0.3*, HOME FAST r=-0.2*.  SR and: Usability A&B r=0.2*, NEWS C r=0.2*, Neighbourhood Satisfaction r=0.4*, HOME FAST r=-0.2*.  LIM and: Usability A r=0.2*, Usability B r=0.3*, NEWS C r=0.2*, Neighbourhood Satisfaction r=0.1, HOME FAST r=^-^0.3*  IR and: Usability A&B r=0.2*, NEWS C r=0.2*, Neighbourhood Satisfaction r=0.2*, HOME FAST r=^-^0.2*  MR and: Usability A&B r=0.2*, NEWS C r=0.1, Neighbourhood Satisfaction r=0.2*, HOME FAST r=^-^0.2*  Overall function and: Usability A&B r=0.2*, NEWS C r=0.1, Neighbourhood Satisfaction r=0.07, HOME FAST r=^-^0.1  UE and: Usability A r=0.03, Usability B r=0.1, NEWS C r=0.1, Neighbourhood Satisfaction r=0.06, HOME FAST r=^-^0.2*  BLE and: Usability A&B r=0.2*, NEWS C r=0.1, Neighbourhood Satisfaction r=0.04, HOME FAST r=^-^0.08  ALE and Usability A&B r=0.2*, NEWS C r=0.1, Neighbourhood Satisfaction r=0.07, HOME FAST Score r=^-^0.1 |
| **Dubuc et al. 2004** | Function (overall, BLE, ALE, UE)  Disability (LIM, FREQ) | The 10-item Physical Functioning Scale of the SF-36 (PF-10) and London Handicap Scale (LHS) | PF-10 and: overall function r=0.85*; BLE r=0.74*; ALE r=0.86*; UE r=0.51*  LHS and: FREQ r=0.47*; LIM r=0.66* |
| **Foster et al. 2011** | Disability  (IR) | Body fat distribution in men and women | IR and total percent body fat: men r=-0.15*, women r=-0.20*  IR and trunk to lower body fat ratio: men r=0.02, women r=0.04 |
| **Franke et al. 2012** | Function  (overall, UE, BLE, ALE) | Objective physical activity, handgrip strength, BMI, Activities of Daily Living Scale (ADL), Bradburn Affect Balance Scale (BABS), Multidimensional fatigue inventory (MFI), self-rated health (SRH) | Physical activity and: overall function r=-0.60*, UE r=-0.70*, BLE r=-0.70*, ALE r=-0.30  Handgrip strength and: overall function r=0.67*, UE r=0.69*, BLE r=0.65*, ALE r=0.66*  BMI and: overall function r=0.35, UE r=0.40, BLE r=0.53*, ALE r=0.15  ADL and: overall function r=-0.66*, UE r=-0.59*, BLE r=-0.68*, ALE r=-0.53*  BABS: (Positive Affect) overall function r=0.76*, UE r=0.59*, ALE r=0.72*, BLE r=0.80*; (Negative Affect) overall function r=-0.24, UE r=-0.51*, BLE r=-0.28*, ALE r=-0.62*  MFI: (Total) overall function r=0.38, UE r=0.64*, BLE r=0.49, ALE r=0.46; (general fatigue) overall function r=0.36, UE r=0.66*, BLE r=0.50, ALE r=0.69*; (physical fatigue) overall function r=0.26, UE r=0.56*, BLE r=0.35, ALE r=0.35; (reduced activity fatigue) overall function r=0.26, UE r=0.36, BLE r=0.31, ALE r=0.26; (reduced motivation fatigue) overall function r=0.58*, UE r=0.65*, BLE r=0.63*, ALE r=0.56*; (mental fatigue) overall function r=0.05, UE r=0.38, BLE r=0.20, ALE r=-0.03  SRH: (overall) overall function r=0.61*, UE r=0.68*, BLE r=0.61*, ALE r=0.70*; (health now vs. 5 years ago) overall function r=0.17, UE r=0.40, BLE r=0.15, ALE r=0.52; (health troubles stand in way) overall function r=-0.47, UE r=-0.85*, BLE r=-0.53*, ALE r=-0.47 |
| **Gibson et al. 2010** | Function (overall)  Disability (FREQ, LIM) | Gender, age group, community services, setting, Beck depression, medical conditions, Timed Up and Go (TUG), pain (WOMAC), self-perceived health status (SPHS), BMI, six-minute walk (6MWT), height, step test, single leg stand, education level | Model including female gender*, age 75-79, age 80-84*, age >85*, no. of community services, retirement dweller, Beck depression*, no. of medical conditions, TUG*, hip pain*, arthritis*, knee pain*, SPHS good, SPHS poor*, BMI*, 6MWT*, and height* predicted overall function with r^2^=0.52  Model including female gender*, Beck depression*, 6MWT*, step test*, SPHS good, SPHS poor*, single leg stand*, hypertension and BMI* predicted FREQ with r^2^=0.20  Model including BMI*, SPHS good, SPHS poor*, live alone*, no. of community services*, secondary education, trade education, university education, no. of medical conditions*, Beck depression*, TUG*, knee pain*, hip pain* predicted LIM with r^2^=0.35 |
| **Hawker et al. 2012** | Disability  (LIM) | A new 11-item Osteoarthritis pain measure (ICOAP) | LIM and ICOAP: r=-0.154 |
| **Hess et al. 2010** | Function (overall)  Disability  (LIM) | Figure-of-8 Walk Test (F8W) | Overall function and F8W: time: r=-0.469*, steps r=-0.348*; smoothness r=0.225  LIM and F8W: time: r=-0.259, steps r=-0.160; smoothness r=-0.052 |
| **Julius et al. 2012** | Function (overall, ALE, BLE)  Disability  (LIM) | Rating of Perceived Exertion (RPE) of walking | RPE and: overall function r=-0.17; BLE r=-0.20; ALE r=-0.11; LIM r=-0.07 |
| **Kafri et al. 2012** | Function (overall, UE, BLE, ALE)  Disability  (LIM, FREQ, IR, MR, SR, PR) | BMI, Incontinence quality of life questionnaire (I-QoL), Visual Analog Scale (VAS) for impact of bladder problems on daily life in women with urinary incontinence (UUI) and controls | Partial correlations after controlling for age and BMI:  BMI (pooled) and: LIM -0.08*, IR -0.07*, MR -0.36, FREQ -0.07, SR 0.01, PR -0.13*, function -0.4*, UE-0.13*, BLE -0.36*, ALE -0.46*  BMI (UUI) and: LIM -0.09*, IR -0.24*, MR -0.03, FREQ -0.07, SR -0.001, PR -0.13*, function -0.40*, UE -0.13*, BLE -0.36*, ALE -0.45*  BMI (continent) and: LIM -0.05, IR -0.08, MR -0.08*, FREQ 0.006, SR 0.06, PR -0.18*, function -0.28*, UE -0.04, BLE -0.14*, ALE -0.28*  I-QoL (UUI) and: LIM 0.29*, IR 0.21*, MR 0.3*, FREQ 0.3*, SR 0.4*, PR 0.1, function 0.14*, UE 0.1, BLE 0.7*, ALE 0.47*  VAS (UUI) and: LIM -0.13*, IR 0.11, MR -0.08*, FREQ -0.23*, SR -0.21*, PR -0.19*, function -0.15*, UE -0.21*, BLE -0.19*, ALE -0.15* |
| **Karp et al. 2009** | Disability  (LIM, FREQ) | Physical Function scale of SF-36 (PF-10), Hamilton Rating Scale for Depression-17, Hamilton Rating Scale for Anxiety, Cumulative Illness Rating Scale, and Mini-Mental State Examination (MMSE) | LIM and: Hamilton Rating Scale for Depression-17 r=-0.38*, Hamilton Rating Scale for Anxiety r=0.41*, PF-10 r=0.47*  FREQ and: PF-10 r=0.35*, MMSE r=0.32*  No significant associations between LIM or FREQ with mental component of SF-36 or Cumulative Illness Rating Scale. |
| **Keysor et al. 2010** | Disability (FREQ, LIM) | Community mobility barriers and transportation facilitators from the Home and Community Environment Survey (HACE) | Unadjusted OR**:**  Community mobility barriers and: LIM 2.2* ; FREQ 1.9*  Transportation facilitators and: LIM: 0.5*; FREQ: 0.8  ORs from model adjusting for age, gender, race, education, BMI, comorbidity, WOMAC function, site and WOMAC pain:  Community mobility barriers and: LIM: 2.0*; FREQ: 1.4  Transportation facilitators and: LIM: 0.5*; FREQ: 0.8 |
| **LaPier 2012** | Function (overall), Disability  (LIM, FREQ) | Physical Activity Scale for the Elderly (PASE), RAND-36 physical functioning subscale, London Handicap Scale (LHS), timed up-and-go (TUG), preferred walking speed (PWS), fast walking speed (FWS), 6-minute walk test (6M), timed sit-to-stand (STS) | Function overall and: PASE r=0.56*, RAND-36 r=0.83*, LHS r=0.65*, TUG r=-0.58*, PWS r=-0.57*, FWS r=-0.55*, 6M r=0.62*, STS r=-0.56*  LIM and: PASE r=0.56*, RAND-36 r=0.68*, LHS r=0.49*, TUG r=-0.26, PWS r=-0.33*, FWS r=-0.24, 6M r=0.33*, STS r=0.12  FREQ and: PASE r=0.54*, RAND-36 r=0.38*, LHS r=0.36*, TUG r=0.00, PWS r=0.01, FWS r=-0.01, 6M r=-0.19, STS r=-0.26 |
| **LeBrasseur et al. 2006** | Disability  (LIM, IR, MR) | Sex, cognition, depression, self-efficacy, and knee extensor strength (model 1) or knee extensor power (model 2) | Model including sex, cognition, depression, self-efficacy, and knee extensor strength (model 1) predicted: LIM r^2^=0.70*, IR r^2^=0.63*, MR r^2^=0.53*  Model including sex, cognition, depression, self-efficacy, and knee extensor power (model 2) predicted: LIM r^2^=0.70*, IR r^2^=0.63*, MR r^2^=0.43* |
| **Li et al. 2012** | Function (overall), Disability  (LIM, FREQ) | Exercise tolerance testing (ETT) protocol, habitual gait speed (HGS), age, sex, weight, medications | Model including ETT*, HGS*, age, sex*, weight and medications predicted overall function with r^2^=0.39*  Model including ETT*, HGS*, age, sex, weight, medications predicted LIM with r^2^=0.20* |
| **Lum et al. 2011** | Function (overall, UE, BLE, ALE) | Acylcarnitine factor scores | Acylcarnitine factor scores and:  Function overall r=-0.195, UE r=-0.049, BLE r=-0.162, ALE r=-0.206 |
| **Melzer & Kurz 2009** | Function  (BLE, UE, ALE)  Disability  (LIM, FREQ) | Berg Balance Scale4 (BBS) and Timed Up and Go (TUG) in non-fallers, fallers and recurrent fallers | Non-Fallers (n=71)  BBS and: function r=0.44,* UE r=0.21, BLE r=0.39,* ALE r=0.48,* LIM r=0.40,* FREQ r=0.25*  TUG and: function r=-0.61,* UE r= -0.3,* BLE r=-0.53,* ALE r=-0.65,* LIM r=-0.51,* FREQ r=-0.36*  Fallers (n=18):  BBS and: function r=0.47,* UE r=-0.18, BLE r=0.35, ALE r=0.62,* LIM r=0.45, FREQ r=0.04  TUG and: function r=-0.67,* UE r=-0.13, BLE r=-0.55,* ALE r=-0.75,* LIM r=-0.44, FREQ r=-0.12  Recurrent fallers (n=11):  BBS and: function r=0.77,* UE r=0.13, BLE r=0.79,* ALE r=0.68,* LIM r=0.60,* FREQ r=0.18  TUG and: function r=-0.62,* UE r=-0.32, BLE r=-0.55, ALE r=-0.55, LIM r=-0.38, FREQ r=-0.14 |
| **Melzer et al. 2007** | Function (overall, UE, BLE, ALE) Disability (FREQ, LIM, SR, PR, IR, MR) | Berg Balance Scale (BBS), Timed Up and Go (TUG) | BBS and: Function overall r=0.48*;UE r=0.30; BLE r=0.51*; ALE r=0.46*; LIM r=0.32; IR r=0.35; MR r=0.15; FREQ r=0.24; SR r=0.15; PR r=0.32  TUG and: Function overall r=-0.52*; UE r=-0.34; BLE r=-0.49*; ALE r=-0.49*; LIM r=-0.26; IR r=-0.30; MR r=-0.19; FREQ r=-0.16; SR r=-0.21; PR r=-0.06 |
| **Newell et al. 2012** | Function (overall)  Disability (FREQ, LIM) | Modified Gait Efficacy Scale (mGES) | mGES and: Function (overall) r=0.88*; FREQ r=0.32*; LIM r=0.63* |
| **Porensky et al. 2009** | Disability  (LIM, FREQ) | Hamilton rating scale for anxiety (HRSA), Generalized Anxiety Disorder severity Scale (GADSS), Penn State Worry Questionnaire (PSWQ) | LIM and: HRSA r=-0.39*; GADSS r=-0.31*; PSWQ r=0.01  FREQ and: HRSA r=-0.30*; GADSS r=-0.22*; PSWQ r=-0.04 |
| **Puthoff & Nielsen 2006** | Disability  (LIM) | Lower-extremity strength, peak power, power at 40%1RM, power at 50%1RM | Simple regression analyses after adjusting for age and sex:  LIM and: Strength r^2^=0.32*; peak power r^2^=035*;power at 40%1RM r^2^=0.31*; power at 50%1RM r^2^=0.22* |
| **Rancourt**  **2009** | Function (overall, UE, BLE, ALE), Disability (FREQ, SR, PR, LIM, IR, MR) | Age, sit and reach (inches), dorsiflexion (ROM), plantarflexion (ROM), knee extension (ROM), knee flexion (ROM), back scratch (inches) | Age and: overall function r=-0.24; UE r=-0.16; BLE r=-0.22; ALE r=-0.23; FREQ r=-0.37*; SR r=-0.40*; PR r=-0.21; LIM r=-0.44*; IR r=-0.44*; MR r=-0.33  Plantarflexion and: overall function r=0.32; UE r=0.35; BLE r=0.38*; ALE r=0.21; FREQ r=0.22; SR r= 0.01; PR r=0.43*; LIM=0.29; IR r=0.28; MR r=0.25  Knee extension and: overall function r=0.18; UE r=0.09; BLE r=0.34; ALE r=0.07; FREQ r=0.40* ; SR r=0.30; PR r=0.41*; LIM r=0.36*; IR r=0.30; MR r=0.39*  Sit and reach and dorsiflexion: all NS with r<0.3  Back scratch and: FREQ r=0.31; PR r=0.32; LIM r=0.34; IR r=0.37* (all others NS with r<0.3)  Regression model including plantarflexion and age predicted: BLE r^2^=0.16; PR r^2^=0.19*; IR r^2^=0.28* |
| **Riddle & Jensen 2013** | Disability  (LIM, FREQ) | Coping strategies questionnaire (CSQ) measures, 7-day pain, Western Ontario and McMasters Universities OA Index (WOMAC), 20m-walk, Center for Epidemeliogic Studies Depression Scale (CESD) | LIM and: CSQ measures r=-0.03(increased behavioural activities) to r=-0.33*(catastrophizing); 7-day pain r=-0.27*; WOMAC r=-0.47*; 20m-walk r=0.37*; CESD r=-0.56*  FREQ and: CSQ measures r=-0.03 (re-interpreting pain) to r=-0.22* (catastrophizing); 7-day pain r=-0.17*; WOMAC r=-0.23*; 20m-walk r=0.24*; CESD r=-0.38*  Hierarchal regression models after adjusting for age, sex, comorbidity, symptom duration, chronic knee pain and BMI:  Model including CSQ catastrophizing*, CSQ ignoring sensations*, CSQ praying and hoping* and CSQ coping self-statements* predicted LIM with r^2^=0.22*  Model including CSQ coping self-statements*, and CSQ catastrophizing* predicted FREQ with r^2^=0.17* |
| **Ritchie**  **2009** | Function (overall) | Berg Balance Scale (BBS), Up & Go (UG) | Baseline overall function and: BBS r=0.73*; UG r=-0.71*  16 weeks, overall function and: BBS: r=0.75*; UG r=-0.71*  *Note: 16-week follow-up was after a falls training intervention.* |
| **Sayers et al. 2004** | Function  (overall, UE, BLE, ALE)  Disability  (LIM, FREQ) | 400-m walk test,  Short Physical Performance Battery (SPPB) | Overall function and: 400-m walk r=0.69*; SPPB r=0.65*  BLE and: 400-m walk r=0.66*; SPPB r=0.63*  ALE and: 400-m walk r=0.73*; SPPB r=0.67*  UE and: 400-m walk r=0.26; SPPB r=0.29  LIM and: 400-m walk r=0.44*; SPPB r=0.37*  FREQ and: 400-m walk r=0.20; SPPB r=0.16 |
| **Segal et al. 2009** | Function  (ALE) | 400-m walk time, SPPB,  gait parameters | ALE and 400 m-walk test: r=-0.56*  ALE and SPPB: r=0.64*  Correlation coefficients between ALE and gait parameters for women all r≥0.25* |
| **Shahar et al. 2009** | Function  (overall, BLE), Disability  (LIM) | Vitamin D metabolites 25(OH)D and 1,25(OH)D, B12, Folate | Overall function and: 25(OH)D r=0.47*; 1,25(OH)D r=0.33*  BLE and: 25(OH)D r=048*; 1,25(OH)D r=0.32*  LIM and: 25(OH)D r=0.31*; 1,25(OH)D r=0.34*  Correlation coefficients for B12 and folate all NS with r<0.2 |
| **Torma et al.**  **2013** | Function (overall) | Age, income, education, BMI, Community Healthy Activities Model Program for Seniors (CHAMPS), Geriatric Depression Scale (GDS-5), Fibromyalgia Impact Questionnaire (FIQR-Pain), Resilience Scale (RS), Medical Outcomes Study-Tangible Social Support Scale (TSS), Charlson Comorbidity Index (CCI) | Model including age*, education*, income*, GDS-5*, BMI*, physical activity*, TSS, and CCI predicted overall Function with r^2^=0.32*  Simple correlations between LLFDI overall function and:  Age r=-0.15*, income r=0.37*, education r=0.22*, BMI r=-0.29*, CHAMPS r=0.20*, GDS-5 r=-0.36*, FIQR-Pain r=-0.54*, RS r=0.32*, CCI r=-0.20*, TSS r=0.0 |

*Significant at p < 0.05

BLE= basic lower extremity scale; ALE=advanced lower extremity scale; UE=upper extremity scale; FREQ=frequency dimension; LIM=limitation dimension; SR=social role domain; PR=personal role domain; IR= instrumental role domain; MR=management role domain

**Table S3.** **Sensitivity to change of the Late-Life Function and Disability Instrument**

| **Study** | **Scales(s)** | **Design** | **Intervention/Assessment interval** | **Between-group results/Effect sizes for within group changes** |
| --- | --- | --- | --- | --- |
| **Adler 2007** | Function  (ALE)  Disability  (LIM, FREQ) | RCT | 10 wks of Tai Chi vs. nonphysical recreational activity | No between-group differences for any outcome measure.  ES for tai chi group: ALE 0.17; FREQ 0.04; LIM 0.60 |
| **Bean et al. 2009** | Function  (overall) | RCT | 16 wks of NIA’s Progressive Resistance Training Program vs. Increased Velocity Exercise Specific to Task (InVEST) program | No between-group difference for any outcome.  Significant time effect for overall function in both groups.  ES InVEST group: 0.24  ES NIA group: 0.09 |
| **Chumbler et al. 2012** | Function  (BLE, UE, ALE)  Disability  (LIM, FREQ, SR, PR, IR, MR) | RCT | 3 months of stroke telerehabilitation intervention vs. usual care | Between-group differences in favour of intervention for: PR (p=0.025), LIM (p=0.025), IR (p=0.031), MR (p=0.024). No other between-group differences.  ES: UE=0.35; BLE=0.44; ALE=0.31; FREQ=0.13; SR=0.01; PR=0.19; LIM=0.66; IR=0.73; MR=0.62 |
| **Clemson et al. 2012** | Function  (overall)  Disability  (LIM, FREQ) | RCT | 12 months of a lifestyle integrated balance and strength training program (LiFE) vs. structured exercise program vs. sham control. | Overall group effect for function (p<0.0001) and FREQ (p=0.01). Between-group differences for LiFE vs. control for function (p<0.0001) and FREQ (p=0.003). Between-group differences for structured program vs. control for function (p=0.04).  ES LiFE group: function=0.41; FREQ=0.26; LIM=-0.10  ES Structured program: function=0.19; FREQ=0.01; LIM=-0.19 |
| **Daniel 2012** | Function  (overall)  Disability  (LIM, FREQ) | RCT | 15 wks of Wii-fit training vs. seated exercise vs. control | No between-group analyses performed.  ES Wii-Fit group: function=0.47; FREQ=0.12; LIM=0.46.  ES Seated Exercise: function=0.32; FREQ=0.22; LIM=0.47. |
| **Davis et al. 2011** | Disability  (FREQ, LIM) | Cohort | Completion of outcome questionnaire 2 wks prior to surgery and 1, 3, 6, and 12 months post total hip (THR) or knee replacement (TKR) | Significant time effect for all outcomes (p<0.0001).  Pre-surgery to 12 months:  ES for THR: FREQ= 0.67; LIM=1.6  ES for TKR: FREQ=0.53;LIM= 0.95  *Note: authors used a 0-10 scale for the LLFDI* |
| **Day et al. 2012** | Function  (overall, BLE, UE, ALE)  Disability  (LIM, FREQ, SR, PR, IR, MR) | RCT | 24 wks of Modified Sun style Tai Chi vs. seated flexibility exercise | No between-group differences for any outcome.  ES all < 0.10, except for SR (ES=0.28) in Tai Chi group. |
| **Demark-Wahnefried et al. 2012** | Function  (ALE, BLE) | Cross-over trial | 12-month diet and exercise intervention (RENEW) followed by 12 months of observation vs. a 12-month delayed intervention | Decreased rates of decline in BLE and ALE in each arm during intervention delivery with an increased rate of decline in the year after intervention completion for the immediate-intervention arm (all p <0.002). |
| **Karp et al. 2009** | Disability  (LIM, FREQ) | Open-label trial | 6 wks of escitalopram treatment for depression | Significant within-group changes for LIM (ES= 0.32, p<0.001) and FREQ (ES=0.18, p<0.001) after 6 wks. In ‘full responders’ ES for LIM=0.81 and FREQ=0.31.  Changes in LIM and FREQ scores after 6 weeks were significantly different between responders and partial/non-responders (both p<0.001). |
| **Keogh et al. 2012** | Function  (overall, UE, BLE, ALE) | RCT | 12 wks of 1x/wk and 2x/wk dance classes vs. controls | Greater improvement in function (overall) and BLE (p<0.05), and a non-significant improvement in UE in favour of 2x/wk vs. control. No between group differences for 1x/wk vs. control.  ES 1x/wk: Function (overall)=0.38; UE=0.39; BLE=.37; ALE=0.27  ES 2x/wk: Function (overall)=0.74; UE=0.57; BLE=0.84; ALE=0.65 |
| **LaPier 2012** | Function  (overall)  Disability  (LIM, FREQ) | Cross-sectional | - | Minimal detectable change (MDC_95_) computed for:  Overall function 4.3  LIM 16.7  FREQ 7.8 |
| **Lenze et al. 2009** | Disability  (LIM) | RCT | 12 wks of escitalopram vs. placebo | Between-group difference in LIM in favour of escitalopram (p=0.04).  ES: LIM=0.32 |
| **Melzer & Oddsson 2012** | Function  (overall, BLE, UE, ALE) | RCT | 12 wks of balance training vs. control | Group by time effect for BLE in favour of training (p=0.006).  ES: BLE=0.49; UE=0.45; ALE=0.32; overall function=0.49  Within-group improvements in overall function in intervention group correlated with step-execution parameters measured under dual-task conditions on force platform (r=-0.49* to -0.56*) |
| **Morey et al. 2009a** | Function  (BLE, ALE) | RCT | 12-month diet and exercise intervention (RENEW) delivered via telephone vs. wait-list control | Between-group effect for BLE (p=0.005) in favour of intervention. Trend for group effect in ALE (p=0.01, adjusted alpha level=0.006).  ES: BLE=0.02; ALE=-0.02  *Note: significant decline in control group (ES: BLE=-0.12, ALE=-0.16)* |
| **Morey et al. 2009b** | Function  (overall)  Disability  (FREQ, LIM) | RCT | 12 months of multicomponent physical activity counseling compared to usual care | No between-group difference for overall function or FREQ. Small between-group difference for LIM (p=0.01) in favour of intervention.  ES: Function=0.04; LIM=0.02; FREQ=0.22 |
| **Ouellette et al. 2004** | Function  (overall, UE, BLE, ALE)  Disability  (FREQ, SR, PR, LIM, IR, MR) | RCT | 12 wks of high-intensity resistance training (PRT) vs. control | Group effect for ALE (p=0.03), LIM (p=0.05) and IR (p=0.05) favouring the PRT group. No other between-group effects.  ES: Function overall=0.72; UE=-0.14; BLE=0.74;ALE=0.78; FREQ=0.67; SR=0.71; PR=0.40; LIM=1.2; IR=1.28; MR=0.48 |
| **Peddle-McIntyre et al. 2012** | Function  (ALE, BLE, UE) | Single-group feasibility study | 10 wks of resistance exercise training | No significant within group changes in any outcome.  ES: ALE=0.13; BLE=0.21;UE=0.14; Overall function =0.16 |
| **Perruccio et al. 2010** | Disability  (LIM) | Cohort | Questionnaires 3 and 6 months post total joint replacement for hip or knee OA | Baseline to 3 months LIM ES=1.22  Baseline to 6 months LIM ES=1.48  Only 4% reported no change in LIM over time. |
| **Richardson et al. 2010** | Function  (overall)  Disability  (FREQ, LIM) | RCT | Multi-component rehabilitation intervention with 6 wks self-management education vs. usual care | No between-group differences over 15 months.  ES from baseline to 15 months: overall function=-0.04; FREQ=-0.09; LIM=0.21 |
| **Ritchie**  **2009** | Function  (overall) | RCT | 16 wks of 1x/wk vs. 2x/wk balance and gait exercise program (FallProof™) | No statistically significant correlations were found between change in LLFDI scores and change in functional tests (BBS, Up and Go). No other results reported for change in LLFDI. |
| **Rosie and Taylor**  **2007** | Function  (overall) | RCT | 6 wks of repeated sit-to-stand (GrandStand System™) vs. progressive resistance knee extension exercises | No significant between-group differences in results for any outcome measure.  ES: GrandStand=0.01  ES: Knee Extension=-0.08 |
| **Segal and Wallace**  **2012** | Function  (BLE) | Single-group study | 6 wks of aquatic power training sessions | No significant within-group changes in BLE after training.  ES at 6 wks=0.20, ES at 12 weeks=0.18 |
| **Travison et al.**  **2011** | Function  (overall) | RCT | 6 months of testosterone administration vs, placebo | No between-group differences in overall function. Minimal important difference (MID) calculated using patient-reported global rating of change was 2.71. No between-group difference in proportion of patients achieving MID in treatment vs. control.  ES overall function=0.31 |
| **VanSwearingen et al. 2011** | Function  (overall, BLE, ALE)  Disability  (LIM, IR) | RCT | 12 wks of task-oriented, motor sequence learning exercise (TO) vs. impairment-oriented exercise (IO) | Between group difference for BLE (p=0.037) in favour of TO. No between-group differences for other variables. Significant within-group change only for BLE (p=0.003, ES=0.38) in TO group.  ES in TO group: Overall function=0.28; ALE=0.16; LIM=0.36; IR=0.47.  ES for IO group all < 0.20. |
| **Winters-Stone et al.**  **2012** | Function  (UE,BLE,ALE)  Disability  (LIM) | RCT | 12 months of resistance and impact training (POWIR) vs. stretching (FLEX) | No between-group differences for UE, BLE, ALE or LIM.  ES POWIR: UE=-0.19; BLE=0.21; ALE=0.07; LIM=0.02 |

ES= effect size; BLE= basic lower extremity scale; ALE=advanced lower extremity scale; UE=upper extremity scale; FREQ=frequency dimension; LIM=limitation dimension; SR=social role domain; PR=personal role domain; IR= instrumental role domain; MR=management role domain
